# The key mechanisms and intervention strategies of cell-type-specific mitochondrial metabolism in ophthalmic diseases

**DOI:** 10.3389/fcell.2026.1961856

**Published:** 2026-09-16

**Authors:** Shouyao Zhang, Xiaochun Yang, Chenggui Xu, Shuang Wang, Xiantao Tai

**Affiliations:** 1 Yunnan University of Chinese Medicine, The First Clinical Medical School, Kunming, China; 2 The First People’s Hospital of Yunnan Province, The Affiliated Hospital of Kunming University of Science and Technology, Department of Ophthalmology, Kunming, China; 3 Yunnan University of Chinese Medicine, The Second Clinical Medical School, Kunming, China

**Keywords:** cell-type-specific, functional disorders, intervention strategies, mitochondria, ophthalmic diseases

## Abstract

The eye is one of the most metabolically active organs, and mitochondria serve as the central hub for cellular energy metabolism by providing adenosine triphosphate through oxidative phosphorylation. Disruption of mitochondrial metabolism—including insufficient adenosine triphosphate synthesis, excessive production of reactive oxygen species, and dysregulated quality control—can trigger various ocular diseases. Common blinding diseases such as myopia, diabetic retinopathy, and glaucoma are closely associated with mitochondrial dysfunction in specific cell types. This review delineates the mechanisms by which metabolic adaptations in scleral fibroblasts induce myopia and the synergistic action of lipofuscin, the high-glucose-triggered mitochondrial reactive oxygen species bursts and metabolic memory in diabetic retinopathy, and the roles of mitochondrial dynamics imbalance and energy exhaustion in trabecular meshwork cells in glaucoma. Based on these mechanisms, the article proposes intervention strategies targeting mitochondrial antioxidant defense, enhancing mitochondrial biogenesis, regulating mitochondrial dynamics, and restoring mitophagy, aiming for precise prevention and treatment of ocular diseases at the mitochondrial level.

## 1 Introduction

The eye, as one of the most metabolically active tissues in the human body, relies on the coordinated function of various cell types including retinal pigment epithelial cells (RPECs), retinal ganglion cells (RGCs), scleral fibroblasts, Iris Smooth Muscle Cells (ISMCs), trabecular meshwork cells (TMCs), and corneal endothelial cells (CECs) to maintain its functions (Jiang et al., 2020). Mitochondria provide the adenosine triphosphate (ATP) required for the physiological functions of these cells through oxidative phosphorylation, and they are also involved in the maintenance of redox homeostasis, calcium buffering, and regulation of apoptosis (Bhatti et al., 2017). Therefore, the stability of mitochondrial metabolism is fundamental to the maintenance of the overall structure and function of the eye. Dysfunction of mitochondria can manifest through various mechanisms in multiple ocular diseases. In diabetic retinopathy, hyperglycemia simultaneously increases flux through the polyol pathway, AGE formation, PKC signaling, and mitochondrial superoxide production. These processe are not linearly connected (Whitehead et al., 2018); rather, they form a self-reinforcing network in which mitochondrial ROS production acts as a major integrating and amplifying mechanism that sustains and propagates cellular damage (Kowluru et al., 2015). In primary open-angle glaucoma (POAG), complex I activity has been found to be impaired, leading to reduced oxidative phosphorylation and ATP production (Va et al., 2015). Subsequent genomic analyses have identified a series of mitochondrial abnormalities in POAG patients, including mutations in genes such as OPA1 and SIX1-SIX6, which can cause mitochondrial dynamics imbalance and selective degeneration of RGCs (Lu et al., 2019; Pan et al., 2024).

However, previous studies have primarily focused on the in-depth elucidation of single diseases or single mechanisms, lacking a unified analytical framework that integrates the specific metabolic characteristics of different cell types across various ocular diseases. These cell-type-specific metabolic characteristics form the deep foundation for the selective involvement of different ocular diseases, but they have not yet been systematically integrated into the core concept of disease mechanisms.

This study begins with the cell-type-specific mitochondrial metabolic characteristics, systematically reviews the mitochondrial physiological properties and energy metabolic requirements of different ocular cells, and elucidates the critical roles of mitochondrial dysfunction in diseases such as myopia and glaucoma. It also proposes advancements in targeted mitochondrial antioxidant therapy, enhanced mitochondrial biogenesis, regulation of mitochondrial dynamics, and restoration of mitochondrial autophagy as intervention strategies, with a view to clinical translation. The aim is to provide a new mechanistic perspective for ocular basic research, and to offer theoretical insights into the underlying reasons for the selective involvement of different ocular diseases in clinical studies.

## 2 The specific roles of mitochondria in different ocular cells

### 2.1 Scleral fibroblasts

The sclera, serving as the outer shell of the eye, is crucial for maintaining the ocular globe’s shape, resisting intraocular pressure, and protecting the ocular contents. The structural integrity and biomechanical strength of the sclera fundamentally determine the stability of the axial length—the parameter that is not only a core determinant of refractive state but also a direct indicator of the progression of pathologic myopia (Harper and Summers, 2015). The biomechanical properties of the sclera are determined by the composition and arrangement of its extracellular matrix (ECM), with type I collagen being the predominant structural protein. The synthesis, secretion, and remodeling of these collagen networks are carried out by fibroblasts distributed within the scleral matri (Gentle et al., 2003).

The collagen synthesis by scleral fibroblasts is an energy-intensive biosynthetic process. The synthesis of collagen precursors occurs in the rough endoplasmic reticulum, and their post-translational modifications, particularly the hydroxylation of proline and lysine, require adequate oxygen molecules, iron ions, and α-ketoglutarate as a co-substrate (Gong et al., 2025); Subsequently, the secretion of procollagen, extracellular processing, and the cross-linking and directional alignment of collagen fibers all depend on continuous energy supply. This energy is primarily provided by mitochondria within the fibroblasts via oxidative phosphorylation. Consequently, the functional state of mitochondria directly determines the scleral fibroblasts’ ability to synthesize and maintain the ECM, thereby influencing the overall biomechanical properties of the sclera. Dysfunction of scleral fibroblasts not only alters cellular behavior and ECM composition but also upregulates matrix metalloproteinases (MMPs), exacerbating ECM degradation and thinning of the sclera (Wu et al., 2022).

The sclera is an avascular tissue, with its nutritional supply mainly relying on the vascular layer of the choroid. This anatomical feature renders the mitochondria of scleral fibroblasts highly sensitive to changes in blood supply. When choroidal blood flow decreases and choroidal thickness diminishes, leading to a prolonged hypoxic microenvironment at the posterior pole of the sclera, it results in reduced collagen expression in scleral fibroblasts (Sun et al., 2024). Recent studies have shown that hypoxia-inducible factor 1α (HIF-1α) is significantly upregulated in myopic scleral tissues and directly participates in the metabolic reprogramming and phenotypic transformation of scleral fibroblasts. Notably, HIF-1α can bind to the hypoxia-response element within the promoter region of the N-acetyltransferase 2 (NAT2) gene, thereby inhibiting its transcriptional activity (Gong et al., 2025). NAT2 is an important metabolic enzyme, recently identified as a gene associated with increased risk of axial length by whole-genome association studies, with its function closely related to mitochondrial metabolism (Choudhury et al., 2024). Therefore, HIF-1α, NAT2, and mitochondrial dysfunction are directly linked to genetic susceptibility and hypoxic microenvironment in scleral fibroblasts (see Figure 1).

**FIGURE 1:**
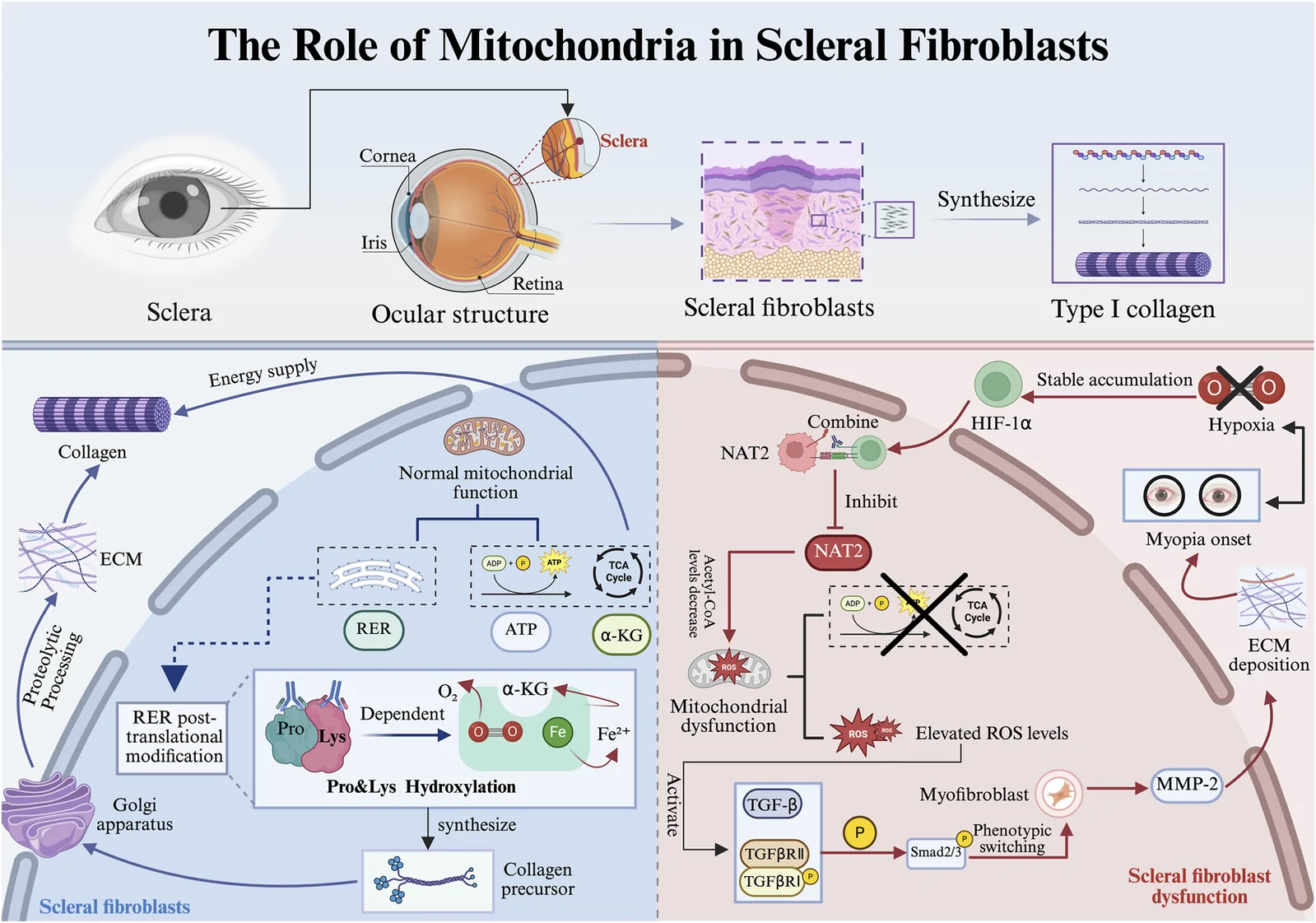
The role of mitochondria in scleral fibroblasts. Type I collagen, synthesized by scleral fibroblasts, is the primary structural protein responsible for maintaining the morphology of the sclera. The energy provided by mitochondria sustains the active metabolic state of scleral fibroblasts, enabling them to secrete a substantial amount of collagen and maintain the biomechanical properties of the sclera. The relative hypoxia in the sclera alters the microenvironment of scleral fibroblasts, leading to mitochondrial dysfunction. This, in turn, accelerates the degradation of the extracellular matrix, ultimately contributing to the development of myopia. Created in BioRender. Xu, C (2026) https://BioRender.com/3eccrwy. Publication license obtained. RER:Rough Endoplasmic Reticulum; ATP: Adenosine Triphosphate; ECM: Extracellular Matrix, α-KG: α-Ketoglutarate; TGF:Transforming Growth Factor; MMP:Matrix Metalloproteinase; NAT: N-acetyltransferase; HIF1:Hypoxia-inducible factor 1.

### 2.2 Iris smooth muscle cells

The iris, as the variable aperture of the anterior segment of the eye, adjusts the pupil diameter through fine-tuned regulation to modulate the amount of light entering the eye, ensuring optimal imaging quality on the retina under different lighting conditions. This function relies on the coordinated contraction and relaxation of two antagonistic smooth muscle groups within the iris—the sphincter pupillae and the dilator pupillae. The contraction and relaxation of smooth muscles are highly energy-consuming processes: the cycling of myosin and actin cross-bridges, the uptake and release of calcium ions by the sarcoplasmic reticulum, and the activities of ion pumps on the cell membrane (such as the Na^+^/K^+^-ATPase) that maintain membrane potential, all depend on the ATP provided by mitochondrial oxidative phosphorylation (Kanda et al., 2016). Research has shown that ISMCs have the highest mitochondrial reserves among all smooth muscle cells. These mitochondria not only provide energy for the rapid contraction of the muscle but also meet the long-term energy demands for maintaining basal tone (Gabella, 2018). These findings suggest that the mitochondrial content of ISMCs may be closely related to their functional requirements, forming the basis for their sustained contraction. While ISMCs are highly mitochondria-dependent, their role in specific ophthalmic diseases remains under-investigated and warrants future research.

### 2.3 Retinal pigment epithelial cells

RPECs are located between the neural retina and the choroid, forming a single-layered polarized epithelium that serves as a core supportive structure for the survival and function of photoreceptor cells. RPECs undertake multiple irreplaceable physiological functions: as components of the blood-retinal barrier, they regulate the directional transport of ions, water, and metabolites; they participate in the visual cycle by re-esterifying all-trans retinal from photoreceptor outer segments and recycling it back to photoreceptors; they daily phagocytose and digest the apical disc membrane of shed photoreceptor outer segments; and they secrete various neurotrophic factors and immunoregulatory factors to maintain the homeostasis of the retinal microenvironment (Ridley et al., 2025). The efficient execution of these functions demands a substantial energy demand. RPECs are rich in mitochondria, which not only generate ATP through oxidative phosphorylation to fuel cellular activities but also provide metabolic intermediates through the tricarboxylic acid (TCA) cycle, regulate cell apoptosis signaling pathways, and participate in lipid metabolism and processing (Tong et al., 2022). Studies have shown that the functional integrity of RPECs is directly related to the health status of their mitochondria, and mitochondrial dysfunction can severely impair RPE function both *in vivo* and in cell culture (Brown et al., 2019). Photoreceptors and Müller cells are also highly metabolically active and closely interact with RPE cells; RPE cells provide metabolic support to photoreceptors through the visual cycle and lactate shuttle, while Müller cells contribute to retinal energy homeostasis by supplying glycolytic substrates to neurons (Kanow et al., 2017; Lindsay et al., 2014). These metabolic interactions are intrinsically linked to mitochondrial function in the RPE-photoreceptor complex.

During the aging process of RPECs, two of the most significant changes are the progressive accumulation of lipofuscin and the decline in mitochondrial energy-generating capacity. Studies have shown that A2E not only a passive accumulation of metabolic waste, can actively damage mitochondrial function. At physiological concentrations, A2E can reduce the mitochondrial membrane potential of RPECs and inhibit the efficiency of oxidative phosphorylation. Under physiological concentrations of glucose or pyruvate, A2E significantly inhibits the phagocytic function of RPECs, and antioxidants can reverse this effect, suggesting that the damage caused by A2E is mediated by oxidative mechanisms (Vives-Bauza et al., 2008). This finding reveals that in the aging process of RPECs, lysosomal waste accumulation and mitochondrial energy depletion are not independent events but rather a mutually reinforcing process, collectively driving the vicious cycle of RPECs functional impairment.

### 2.4 Trabecular meshwork cells and corneal endothelial cells

Trabecular meshwork cells (TMCs) and corneal endothelial cells (CECs) serve as key cellular populations in the anterior chamber, respectively regulating aqueous humor outflow resistance and maintaining corneal deturgescence, two core physiological functions that are highly dependent on mitochondrial energy metabolism. However, their energy requirements and metabolic characteristics differ. Trabecular meshwork cells are located in the anterior chamber angle, where they actively phagocytose pigment particles and metabolic debris from the aqueous humor and modulate aqueous humor outflow resistance through the dynamic remodeling of the extracellular matrix. Both of these core functions are energy-intensive active processes that require adequate ATP production from mitochondrial oxidative phosphorylation to support cytoskeletal remodeling, vesicle trafficking, and lysosomal degradation (Saccà et al., 2016). Advances have also been made in the mitochondrial dynamics regulatory mechanisms of TMCs. Recent studies indicate that chronic stress-induced elevation of endogenous glucocorticoids can promote trabecular meshwork fibrosis and mitochondrial dysfunction, with molecular mechanisms involving upregulation of monoamine oxidase A (MAOA) and abnormal activation of mitochondrial fission. Knockdown of MAOA or mitochondrial elongation factor 1 (MIEF1) significantly reduced mitochondrial fragmentation and improved cellular function (Sun et al., 2026), suggesting that the MAOA-MIEF1 signaling axis is a key pathway in regulating the mitochondrial dynamics homeostasis of TMCs.

The cornea is an energy-intensive tissue that is highly sensitive to external stimuli. During the repair process, CECs require rapid ATP turnover to promote cell proliferation and migration. The Na^+^/K^+^-ATPase on the basolateral membrane of CECs actively pumps intracellular Na^+^ into the aqueous humor side, establishing a permeability gradient that drives water molecules through aquaporins from the corneal stroma. This active transport process consumes a large amount of ATP, which is met through increased mitochondrial numbers and enhanced mitochondrial calcium uptake (Hatou, 2011). Recent mechanistic studies have shown that activation of the COL8A2 gene can increase phosphorylated YAP levels through the Hippo signaling pathway, promoting ATP production, enhancing mitochondrial membrane potential, and thereby improving the barrier and pump functions of CECs (Ryu et al., 2024). Indeed, mitochondria interact with CECs through multiple biological pathways such as autophagy (McWilliams et al., 2019), inflammation (Li et al., 2024), and apoptosis (Shan and Fan, 2016), due to the dynamic nature of mitochondria, which rely on fission-fusion dynamics to adapt to external changes. Mitochondrial redox signaling perturbations exacerbate oxidative stress and the release of mitochondrial-derived damage-associated molecular patterns, triggering inflammatory cascades and cell death pathways. Disruption of mitochondrial dynamics in the cornea further exacerbates cellular damage and disease progression (Yang et al., 2026). Targeting mitochondria in the treatment of corneal diseases holds significant potential.

## 3 The core mechanisms of mitochondrial metabolic disorders in different cell types in ocular diseases

### 3.1 Scleral fibroblasts and myopia

Myopia, especially pathologic myopia, is widely regarded as a disease associated with structural changes in the posterior pole of the eye, characterized by excessive elongation of the eye axis. Earlier studies on myopia pathogenesis have primarily focused on optical defocus and genetic susceptibility, whereas recent evidence has increasingly implicated metabolic and mitochondrial dysfunction as important contributors to scleral remodeling (Yu and Zhou, 2022).

Scleral hypoxia is a common feature of myopia, primarily caused by reduced choroidal blood supply (Shi et al., 2025). Thinning of the choroidal capillary layer and reduced blood flow result in a relatively chronic hypoxic microenvironment in the posterior pole of the sclera (Zhang et al., 2025). Under hypoxic conditions, HIF-1α upregulates MMPs, promoting ECM remodeling and degradation, leading to scleral thinning and axial elongation, and initiating a series of downstream gene expressions (Ren Y. et al., 2022; Zhao et al., 2020). By reducing HIF-1α levels, these processes can be alleviated, thereby inhibiting the progression of myopia (Wen X. et al., 2025; Wu et al., 2018). The most direct and fundamental impact of hypoxia on cells lies in the mitochondria. Studies have shown that mitofusin 2 (Mfn2) plays a critical role in maintaining the homeostasis of scleral fibroblasts. In a myopia rat model, hypoxia-induced expression of Mfn2 in scleral fibroblasts is significantly reduced. Overexpression of Mfn2 can maintain endoplasmic reticulum (ER) homeostasis, inhibit fibroblast-to-myofibroblast transdifferentiation induced by hypoxia, upregulate the expression of collagen α1 chain, reduce ER stress and mitochondrial damage, and decrease cell apoptosis (Wang Y. et al., 2025).

Under sustained hypoxic stress, the energy metabolism of scleral fibroblasts undergoes a transformation, forcing the cells to shift from efficient ATP production via oxidative phosphorylation to inefficient glycolysis, which does not require oxygen. The increase in scleral glycolysis-lactate levels promotes Notch1 expression, which may be associated with the significant enrichment of genes encoding glycolytic enzymes, such as ENO2 and TP11 (Lin et al., 2024). This shift in energy metabolism suppresses mitochondrial biogenesis, weakening the cell’s long-term energy reserves and stress resistance. In this context, hypoxia and metabolic disorders induce the mitochondria to produce excessive and destructive ROS. These ROS activate multiple signaling pathways, including p38 MAPK (Ren et al., 2022), PI3K-AKT, NF-κB (Chen et al., 2025) and Wnt/β-catenin (Wen B. et al., 2025), which in turn upregulate the expression of MMP-2. MMP-2 is an enzyme crucial for ECM degradation, and studies have shown that MMP-2 activity is elevated in the sclera of myopic eyes, leading to increased degradation of collagen and other ECM components. This enhanced activity results in scleral thinning and elongation, which are key factors in myopia progression (She et al., 2022; Wahyuningsih et al., 2022).

In summary, mitochondrial metabolic dysfunction is an important metabolic contributor to scleral remodeling during myopia development, the scleral remodeling during myopia development is essentially a disturbance in mitochondrial metabolic homeostasis of fibroblasts under a hypoxic microenvironment. It is important to note that myopia is multifactorial and also involves retinal signaling, choroidal responses, optical defocus (Zhang et al., 2026), and genetic susceptibility (He and Li, 2023), and mitochondrial dysfunction is one component of this complex network. In the future, by developing therapies that improve mitochondrial function and target the regulation of MMP activity, the target can be extended from the optical system of the eye to the cellular metabolic system, potentially delaying or even halting the progression of pathologic myopia.

### 3.2 Retinal microvascular endothelial cells and diabetic microvascular lesions

Diabetic retinopathy (DR) is the most common microvascular complication of diabetes, DR risk depends on multiple factors including diabetes duration, glycemic exposure, blood pressure, and ethnicity (Nentwich and Ulbig, 2015). Recent studies have defined DR as a progressive functional impairment of the “neurovascular unit,” which encompasses photoreceptors, bipolar cells, ganglion cells, Müller cells, pericytes, astrocytes/microglia, and microvascular endothelial cells (Ren J. et al., 2022; Nian et al., 2021). This unit’s functional coupling is highly dependent on a stable energy supply. For instance, RMECs play a crucial role in the pathophysiology of DR. Dynamin-related protein 1 (Drp1), a GTPase dynamics-related protein, is closely associated with microvascular damage induced by hyperglycemia. Chen et al. discovered that under high glucose conditions, Drp1 levels significantly increase, exacerbating mitochondrial damage and apoptosis rates in RMECs, thereby contributing to the onset of DR (Chen et al., 2023). Evidence from lipidomics and network pharmacology further indicates that sphingosine and sphingosine-1-phosphate intensify the damage to diabetic rat RMECs induced by high glucose, increasing MDA and ROS levels, and that the AGE-RAGE and HIF-1 signaling pathways, along with the reduction in OPA1 levels due to cellular apoptosis, may be involved in this process (Kim and Roy, 2020; Yuan et al., 2023).

In diabetic states, persistent hyperglycemia leads to excessive glucose influx into cells, overloading the mitochondrial tricarboxylic acid cycle flux, with complex I and III of the, ETC., becoming bottlenecks for electron leakage, directly transferring excess electrons to oxygen molecules, thereby generating excessive ROS (Che et al., 2026). These ROS, in turn, enhance the formation of polyol pathways, PKC, and AGEs (He et al., 2025). These pathways form a complex interplay network: ROS activate PKC, which in turn activates NADPH oxidase, leading to the production of even more ROS; AGEs interact with their receptor RAGE (Kassab and Piwowar, 2012), continuously stimulating the mitochondria to produce more ROS. This self-reinforcing positive feedback loop constitutes the metabolic memory cycle of DR (Kowluru and Alka, 2023), resulting in mitochondrial fragmentation. Even when subsequent glucose levels return to normal, early high-sugar exposure-induced retinal microvascular damage continues to progress. However, this ROS-PKC-AGE feedback loop is only one component of the broader concept of metabolic memory, which also encompasses persistent mitochondrial DNA damage, DNA methylation and histone modifications (Kowluru, 2013), impaired mitophagy (Kowluru et al., 2024), chromatin remodeling (Mohammad et al., 2026), inflammatory priming (Kowluru, 2023), and metabolic reprogramming (Lv et al., 2022). Chronic metabolic stress and oxidative damage cause the retina to enter a state of low-grade inflammation. This inflammatory response further exacerbates vascular leakage, attracts leukocyte adhesion, and inversely inhibits mitochondrial function. Therefore, it is particularly important to alter the inflammatory environment of the retina. Research has shown that the Nuclear factor erythroid 2-related factor 2 (Nrf2) activator DH404 can prevent retinal and vitreous cavity vascular leakage, suppress the upregulation of inflammatory mediators such as TNF-α and IL-6, and maintain the integrity of Müller cell gliosis, thereby preventing retinal barrier dysfunction (Deliyanti et al., 2018) (see Figure 2).

**FIGURE 2:**
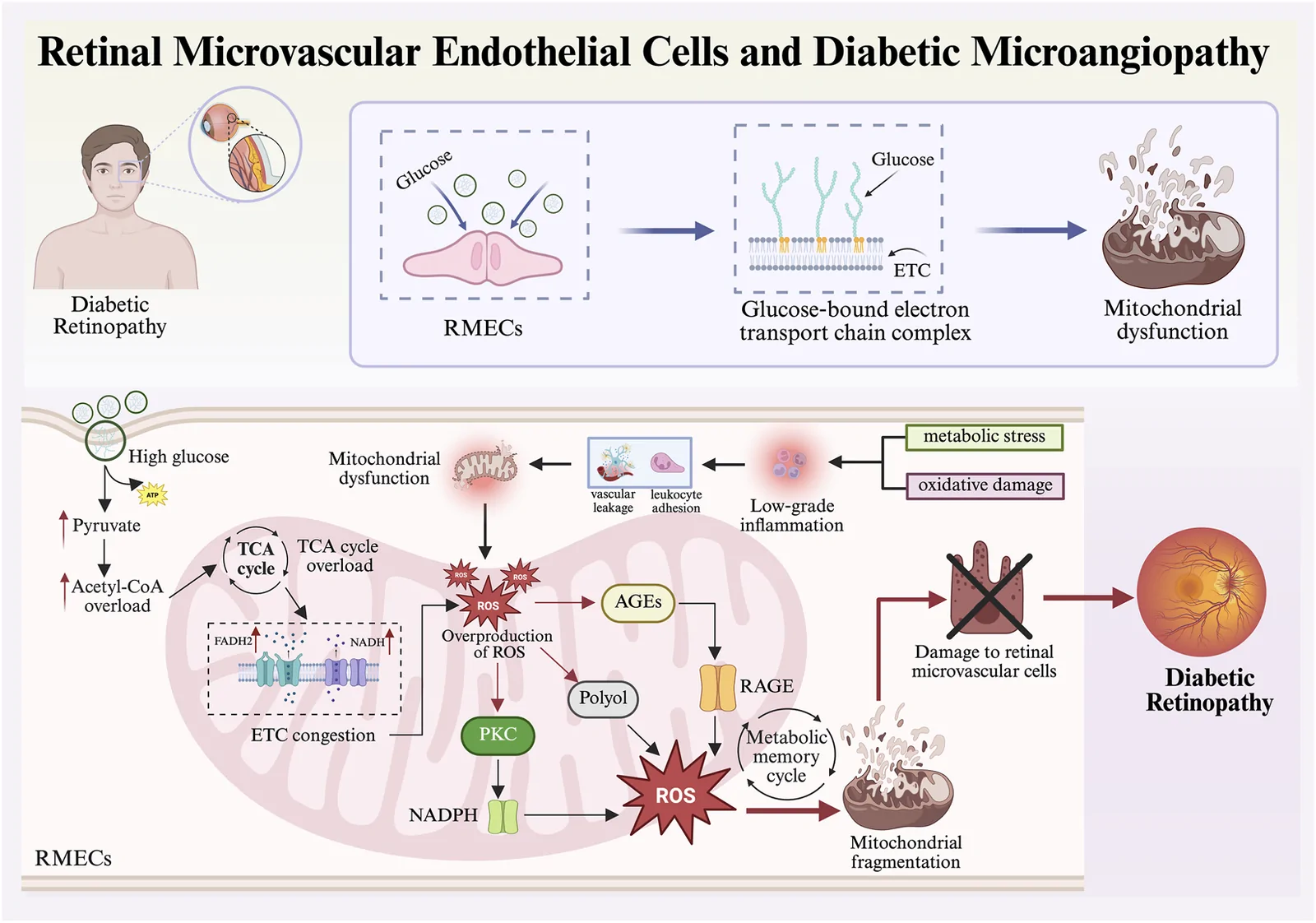
Retinal Microvascular Endothelial Cells and Diabetic Microangiopathy. Diabetic retinopathy is one of the common microvascular complications of diabetes. Persisted hyperglycemia disrupts the tricarboxylic acid cycle, leading to excessive production of reactive oxygen species; this leads to mitochondrial dysfunction and damage to retinal microvascular endothelial cells, ultimately resulting in diabetic retinopathy. Created in BioRender. Xu, C (2026) https://BioRender.com/08v8l9n. Publication license obtained. RMECs: Retinal Microvascular Endothelial Cells; ROS: Reactive Oxygen Species; NADPH: Nicotinamide Adenine Dinucleotide Phosphate; RAGE:Receptor for Advanced Glycation End-products; ETC: Electron Transport Chain.

In summary, retinal microvascular cells in the retina exhibit a special sensitivity to mitochondrial dysfunction induced by high glucose, with the high-glucose environment disrupting mitochondrial homeostasis and triggering a series of cascading reactions, ultimately leading to the development of diabetic retinopathy. This provides a new perspective for understanding the pathological progression of diabetic retinopathy.

### 3.3 Retinal ganglion cells, trabecular meshwork cells and glaucoma

Glaucoma, the second leading cause of irreversible blindness globally, affects an estimated 80 million people. With the global aging population, it is projected that by 2040, glaucoma will impact approximately 112 million individuals (Tham et al., 2014). The core pathology of glaucoma involves the progressive loss of RGCs and their axons. Given that RGCs possess long axons extending from the eye to the brain, they are highly dependent on the energy generated by oxidative phosphorylation to transmit visual signals, making them more susceptible to mitochondrial dysfunction (Kang et al., 2021; Wareham et al., 2022).

Under physiological conditions, approximately half of the mitochondria within axons are in a quiescent state, providing a stable local energy supply, while the remaining mitochondria undergo anterograde or retrograde transport to update the mitochondrial network and remove damaged mitochondria (Jeong et al., 2025). In glaucoma, elevated intraocular pressure-induced mechanical stress can directly compress axons, impeding mitochondrial axoplasmic transport (Sun et al., 2025). Live imaging studies have provided direct evidence for this mechanism; In a *Xenopus* optic nerve model expressing glaucoma-related OPTN mutant alleles, the proportion of quiescent mitochondria within axons significantly increases, and a large number of mitochondria are excluded from axons and phagocytosed and degraded by surrounding astrocytes (Jeong et al., 2025). This reveals that in glaucoma, not only is mitochondrial transport impeded, but there is also non-autonomous clearance of mitochondria, leading to RGC death.

TMCs are located in the anterior chamber angle, covering the trabecular mesh, and through their active phagocytic function, they clear pigment particles and metabolic debris from the aqueous humor. They also regulate the outflow resistance of aqueous humor by dynamically remodeling the extracellular matrix (Saccà et al., 2015). Both of these core functions are highly energy-intensive active processes that require sufficient ATP from mitochondrial oxidative phosphorylation to support cytoskeletal remodeling, vesicular transport, and lysosomal degradation (He et al., 2008). The mitochondrial functional state of TMCs directly determines their ability to maintain the patency of the aqueous humor outflow pathway. Compared to normal TMCs, TMCs derived from glaucoma exhibit significantly reduced NAD^+^ levels, impaired mitochondrial phagocytic function, and reduced oxidative respiration. Exogenous NAD^+^ supplementation can effectively restore the mitochondrial function and cellular activity of TMCs (Liu et al., 2024). In summary, the unique metabolic vulnerability of RGCs and their long axons, as well as the mitochondrial aging of TMCs in the aqueous humor outflow pathway, together form the deep metabolic basis for the occurrence and development of glaucoma (see Table 1).

**TABLE 1:** Cell-type-specific mitochondrial phenotypes and therapeutic strategies.

Cell type	Mitochondrial phenotype	Associated disease	Key pathway	Mitochondria-targeted therapeutic strategy	Level of evidence
Scleral fibroblasts	Relies on oxidative phosphorylation to provide ATP for collagen synthesis, secretion, and fiber cross-linking; mitochondria are highly sensitive to local hypoxia and reduced blood supply	Pathologic myopia; axial elongation	HIF-1α/NAT2 signaling axis; mitophagy-related pathways; Nrf2 antioxidant pathway	Improving local scleral hypoxia; correcting HIF-1α/NAT2 pathway abnormalities; protecting mitochondrial function in scleral fibroblasts	Low (basic research)
Iris smooth muscle cells	Abnormal mitochondrial morphology (enlargement, cristae disruption, crystalline inclusions), leading to energy failure and cellular dysfunction	Pupillary dysfunction; MELAS syndrome; congenital iris defects	Drp1-mediated dynamics pathway; Nrf2 antioxidant pathway	Mitochondrial antioxidants; mitochondrial biogenesis enhancers	Low (basic research)
Retinal pigment epithelial cells	Impaired oxidative phosphorylation and reduced mitochondrial energy-generating capacity	Age-related macular degeneration; retinitis pigmentosa	Mitochondrial ROS-oxidative stress axis; Nrf2 antioxidant pathway; mitophagy pathway; Drp1-mediated mitochondrial dynamics and SOX-related fibrotic pathways	Mitochondrial antioxidants; mitochondrial biogenesis enhancers; mitophagy inducers	Low (basic research)
Trabecular meshwork cells	Mitochondrial swelling, cristae disruption, impaired oxidative phosphorylation, ATP deficiency, excessive ROS release, and accumulation of senescent cells	Glaucoma	Nrf2 antioxidant pathway; PINK1/Parkin-mediated mitophagy pathway	Mitochondrial antioxidants; mitophagy inducers	Low (basic research)
Retinal endothelial cells	Disruption of mitochondrial structure and impaired oxidative phosphorylation	Diabetic retinal microvascular disease	Nrf2 antioxidant pathway; Drp1-related pathways; PINK1/Parkin-mediated mitophagy pathway	Mitochondrial dynamics regulators	Low (basic research)
Retinal ganglion cells	Mitochondrial swelling and cristae fragmentation in both Soma and axons; impaired mitophagic flux	Glaucoma; diabetic optic neuropathy; hereditary optic neuropathies	Nrf2 antioxidant pathway; AMPK pathway; PINK1/Parkin-mediated mitophagy pathway	Mitochondrial antioxidants; mitochondrial biogenesis enhancers; mitochondrial dynamics regulators; mitophagy inducers	Low (basic research)

## 4 Targeted therapeutic strategies for mitochondria

Mitochondria-targeted therapeutic strategies hold considerable promise for restoring cellular homeostasis and preserving ocular function. However, much of the current evidence remains preclinical, and translation to clinical practice faces several challenges including delivery barriers, target specificity, and safety concerns, which necessitate further in-depth exploration and validation through large-scale clinical studies.

### 4.1 Mitochondrial antioxidants

RGCs: Mitochondria are the primary sites of ROS production within the cell, and conventional antioxidants often struggle to effectively enter the mitochondrial matrix. Therefore, developing antioxidants that can selectively accumulate within mitochondria represents a primary strategy. Nrf2 is a crucial transcription factor that activates various antioxidant pathways, including glutathione, to exert antioxidant effects on RGCs. Another important regulator of the antioxidant defense system is Matrine (MAT), a bioalkaloid derived from Sophora flavescens. MAT activates SIRT1, which in turn upregulates PGC-1α and Nrf2, promoting mitochondrial biogenesis and reducing oxidative stress in RGCs (Song et al., 2022). Studies have shown that SIRT1 can prevent the loss of RGCs in multiple acute and subacute optic neuropathy models by balancing mitochondrial biogenesis and mitophagy, and enhancing mitochondrial self-repair, which may induce neuroprotection (Chaqour et al., 2025).

RPECs: As semi-autonomous organelles, mitochondria have their own unique genome, separate from nuclear genes, such as 12 S rRNA and 16 S rRNA, which contain short open reading frames capable of transcription and translation to synthesize short peptides that regulate cellular activities, known as MDPs. Humanin is the first MDP discovered, which not only plays critical protective roles in the nervous, cardiovascular, and metabolic systems but also protects RPECs from oxidative stress, enhances mitochondrial respiration frequency and biogenesis, and reduces apoptosis (Nashine and Kenney, 2024). Sappanone A (SA) is a naturally occurring flavonoid derived from Sophora japonica, known for its antioxidant activity. Recent evidence indicates that SA alleviates retinal oxidative stress, restores mitochondrial function, and inhibits inflammation and apoptosis in diabetic retinopathy (DR) via activation of the SIRT7/NRF2 signaling pathway (Wang et al., 2026). Overall, antioxidant therapies are critical in maintaining mitochondrial metabolic function (see Figure 3).

**FIGURE 3:**
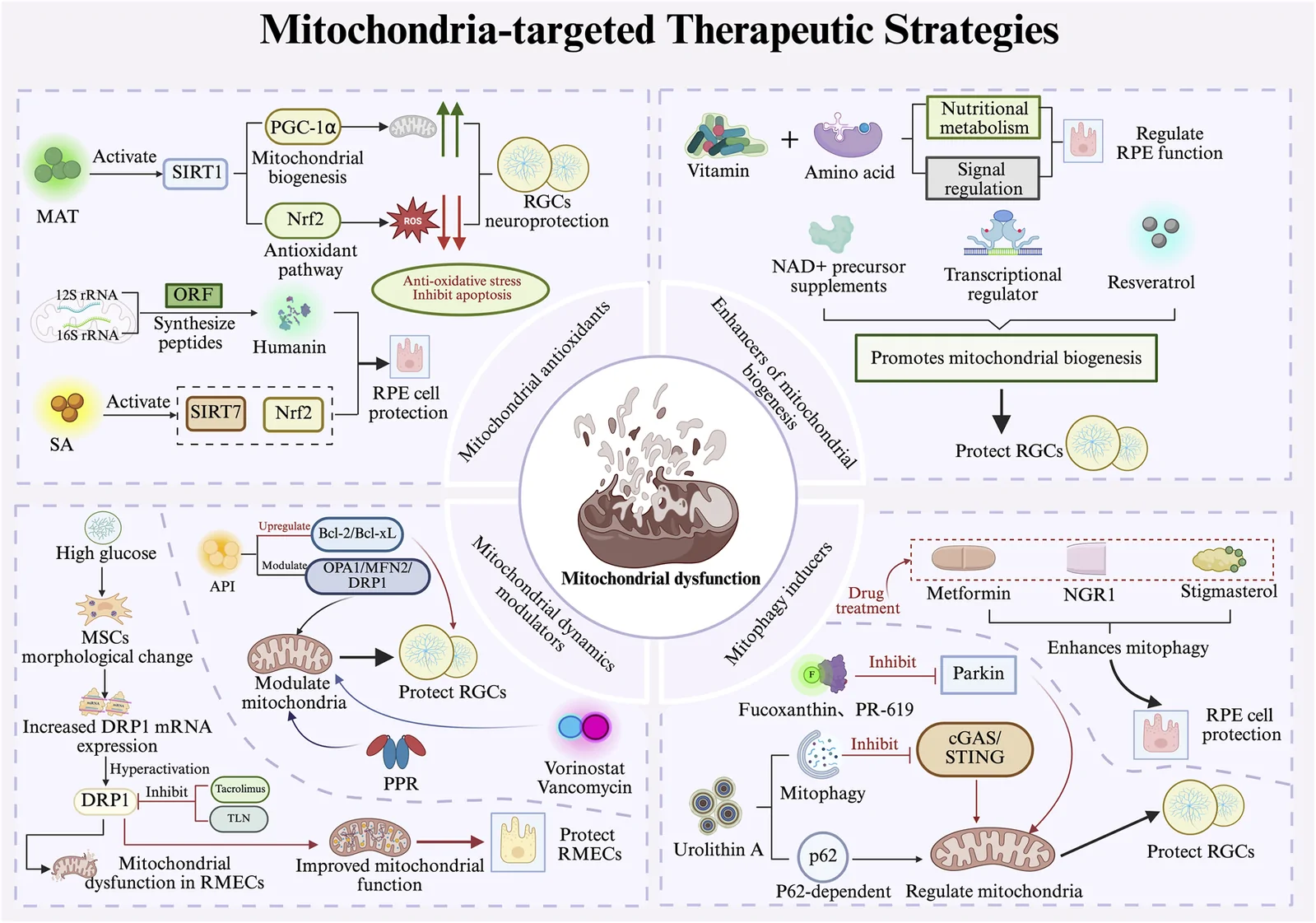
Mitochondria-targeted Therapeutic Strategies. Mitochondria serve as the energy centers for retinal cells, where retinal ganglion cells, retinal pigment epithelial cells, and retinal endothelial cells contain a high significant number of mitochondria. Targeting mitochondrial function holds great potential for the treatment of ophthalmic diseases. Mitochondrial antioxidants, mitochondrial biogenesis enhancers, mitochondrial dynamics regulators, and mitophagy inducers all act from various angles to modulate mitochondrial function, thereby protecting various types of ocular cells and preventing ophthalmic diseases. Created in BioRender. Xu, C (2026) https://BioRender.com/7bvp3zw. Publication license obtained. RGCs: Retinal Ganglion Cells; RPE:Retinal Pigment Epithelium; RMECs: Retinal Microvascular Endothelial Cells; MAT: Matrine; API:Apigenin; ORF: Open Reading Frame; PPR:Proton-pumping rhodopsin; Nrf:Nuclear factor erythroid 2–related factor; DRP1:Dynamin-related protein 1; NGR1:Notoginsenoside R1; cGAS/STING: cyclic GMP-AMP synthase/stimulator of interferon genes; SIRT:Sirtuin; SA:Sappanone A.

### 4.2 Mitochondrial biogenesis enhancers

RGCs: Mitochondrial biogenesis refers to the process by which cells generate new, fully functional mitochondria. Studies have shown that resveratrol can activate the AMPK/PGC-1α signaling pathway, upregulate the expression of Nrf-1, Tfam, Mfn2, and OPA1, and reduce apoptosis, mitochondrial membrane depolarization, and ROS production in RGCs. In a rat model of chronic ocular hypertension, resveratrol has been demonstrated to improve protein expression and mitochondrial dynamics (Zhang et al., 2018). Further research has confirmed that resveratrol enhances mitochondrial biogenesis by acting on key effectors such as SIRT1, ERRs, TERT, TFAM, and Nrf-2. However, it should be noted that these findings are supported primarily by non-ocular cell studies (Chuang et al., 2019; Shaito et al., 2023). NAD^+^ precursors are currently among the most extensively studied mitochondrial biogenesis enhancers. NAD^+^ serves as a cofactor for deacetylases such as SIRT1, and its intracellular level directly determines the efficiency of SIRT1’s deacetylation activation of PGC-1α. Research has found that NAD^+^ levels in the retina of glaucoma model mice decline with age, and oral administration of nicotinamide can protect RGC dendritic structures, significantly delaying retinal dysfunction (Cimaglia et al., 2024). Transcriptional regulators are another important class of mitochondrial biogenesis enhancers. Retinal ganglion cell loss is a characteristic of optic neuropathy, and human embryonic stem cells (hESCs) are a cell source for generating human RGCs *in vitro* (Visalli et al., 2026). Gill et al. generated enriched RGC populations from hESCs through progressive retinal differentiation, finding that enriched hESC-RGCs exhibit longer axons and functional electrophysiological characteristics, and that mitochondria in enriched hESC-RGCs undergo bidirectional axonal transport (Gill et al., 2016). This suggests that mature RGCs maintain their homeostasis by transporting mitochondria via axons.

CECs: Carotenoids exhibit mitochondrial protective effects in the cornea as well. Cloquintocet-mexyl reduces UV-A-induced damage to CEpCs by promoting mitochondrial biogenesis, maintaining mitochondrial morphology, and function (Otsu et al., 2022). Other carotenoids such as astaxanthin and lutein activate mitochondrial biogenesis via AMPK-dependent pathways, as demonstrated in certain non-ocular cell models under oxidative stress.

RPECs: Vitamins and amino acids play crucial roles in regulating the function of RPECs. Shen et al. elucidated that a combination therapy with vitamin C and valproic acid can activate the EMT in fetal RPE-like stem cells (fRPESCs) via SOX2/TGF-β1/SNAIL1 and SOX2/SNAIL1/catenin-ε pathways (Shen et al., 2020), providing new insights into the application of cell therapy in ocular diseases.

### 4.3 Mitochondrial dynamics regulators

Retinal endothelial cells (RECs): Regulatory factors for mitochondrial fission—dynamin-related protein 1 (DRP1) is often overactivated. Studies have shown that by inhibiting DRP1, the mitochondrial volume in retinal microvascular endothelial cells is restored, which in turn restores the integrity of the blood-retinal barrier and improves the vision of glaucoma mice (Cueva Vargas et al., 2026). Mitochondrial dysfunction in retinal microvascular endothelial cells is also a key driver in diabetic retinopathy. Studies have demonstrated that inhibition of Drp1-mediated mitochondrial fission significantly alleviates high-glucose-induced retinal endothelial cell injury, restores mitochondrial morphology, and attenuates the increase in retinal vascular permeability (Chen et al., 2023). S. miltiorrhizais is widely used in some Asian countries as a treatment for cardiovascular diseases. Tanshinones IIa (TanIIa) is one of the main lipophilic components of S. miltiorrhizais. Qian et al. found that TanIIa treatment not only effectively alleviates oxidative stress in RECs but also suppresses mitochondrial fission induced by methylglyoxal (MGO) exposure, increasing the mRNA levels of OPA1 and Mfn1 in retinal endothelial cells (Qian et al., 2019).

RPECs: Emerging mitochondrial dynamics regulatory strategies have made initial progress. HDAC inhibitors such as Vorinostat and Trichostatin A have been shown to enhance mitochondrial fusion, activate the Nrf2 pathway, and repair the cytoskeleton under oxidative stress conditions, thereby protecting RPECs (Lugassy et al., 2025). This provides a new perspective on how epigenetic regulation can influence mitochondrial dynamics.

RGCs: Apigenin (API), a member of the flavonoid subgroup, is found in vegetables, fruits, herbs, and plant-based beverages (Hostetler et al., 2017). In the retinal ischemia/reperfusion (RIR) model, API not only upregulates the expression of Bcl-2 and Bcl-xL, thereby reducing RGC apoptosis, but also regulates the expression of OPA1, Mfn2, and Drp1 to restore mitochondrial morphology (Wu et al., 2024), thus modulating the mitochondrial dynamics of damaged RGCs in the RIR model. More advanced optogenetic strategies can precisely regulate mitochondrial membrane potential. Proton-pumping rhodopsin (PPR) is a widely existing photosynthetic system in nature that utilizes light energy to generate an outward proton flux, driving relevant cellular processes (Claassens et al., 2013). Chen et al. recently reported that a mitochondria-targeted PPR—mt-EcGAPR—can increase mitochondrial metabolism and significantly enhance ATP production under environmental light conditions, thereby protecting retinal ganglion cells in a high intraocular pressure model (Yang et al., 2025). Although this technology primarily functions by directly regulating proton pump activity rather than mitochondrial fission and fusion, mt-EcGAPR represents a promising therapeutic approach that can provide important insights into the treatment of glaucoma and mitochondrial-related neurodegenerative diseases through optogenetic regulation of mitochondrial energy. However, this strategy is currently hypothetical in ophthalmic disease, and further validation in ocular models is needed. In summary, selecting the most appropriate intervention strategies based on the dynamic characteristics of different cell types is becoming a key direction for precise treatment.

### 4.4 Mitophagy inducers

Mitophagy, as a core quality control mechanism for selectively removing damaged mitochondria, plays an indispensable role in maintaining the homeostasis of highly metabolically active cells such as retinal pigment epithelial cells (RPECs), retinal ganglion cells (RGCs), and vascular endothelial cells (Jiménez-Loygorri et al., 2023). Consequently, restoring or enhancing mitophagy function has become a forefront area of intervention research in ocular diseases.

RGCs: Urolithin A, a gut microbiota metabolite of ellagic acid, can induce a reduction in cGAS/STING activation, lower the level of mitochondrial DNA in RGCs, and exhibit significant protective effects in retinal degenerative disease models (Jimén et al., 2024). Additionally, urolithin A can promote lysosomal function via a p62-dependent pathway, reduce the accumulation of damaged mitochondria, and enhance the clearance of damaged mitochondria (Jiménez-Loygorri et al., 2024; Venkatesan and Bernstein, 2025). Fucoxanthin, a carotenoid found in brown algae, diatoms, and certain dinoflagellates, Ma et al.found that intravitreal administration of fucoxanthin during acute intraocular pressure elevation inhibited Parkin expression and excessive mitophagy, increased the survival rate and Bcl-2 expression of RGCs, and reduced the acidic levels of Bax and glial fibrillary acidic protein (GFAP). This study underscores the importance of mitophagy balance; excessive mitophagy is also detrimental to the activity of RGCs (Ma et al., 2025). Recently, deubiquitinating enzyme (DUB) inhibitors, such as PR-619, have shown potential neuroprotective effects by regulating Parkin-mediated mitophagy in RGCs. In glaucoma models, PR-619 upregulated Parkin expression, increased the LC3-II/LC3-I ratio, and elevated the levels of LAMP1. Additionally, in the optic nerves of rats subjected to elevated intraocular pressure, PR-619 treatment increased the number of autophagosomes (Hu et al., 2024). These studies suggest that enhancing Parkin-mediated mitophagy is a feasible therapeutic approach for glaucomatous neurodegeneration.

RPECs: In recent years, metformin has shown anti-inflammatory and antioxidant potential in RPECs through mitochondrial induction. Studies have found that metformin reduced the accumulation of autophagic substrates such as SQSTM1/p62 and the levels of LC3I and II after exposure to antimycin A, thereby improving the mitophagy function of RPECs (Toppila et al., 2024). Notoginsenoside R1 (NGR1) is a novel ginsenoside extracted from Panax notoginseng. Zhou et al.discovered that NGR1 upregulated the levels of PINK1 and Parkin in rMC-1 cells and db/db mouse RPECs, increased the LC3-II/LC3-I ratio, and suggested that NGR1 could prevent diabetic retinopathy (DR) by enhancing mitophagy. The latest research further confirms the potential application of mitophagy inducers in ocular diseases (Zhou et al., 2019). Qin et al.discovered via RNA-seq that stigmasterol enriched the gene expression of mitophagy-related GO items in RPECs, while reducing the expression of VAMP7, a mitophagy-related gene (Wang Q. et al., 2025). These findings provide new insights for the treatment and drug development of DR. They indicate that mitophagy induction is a strategy to reduce age-related inflammation and extend healthy lifespan. Given the predominantly preclinical nature of these strategies, Table 2 summarizes their potential clinical applications, lifestyle-related modulators, current limitations and the key challenges for translation (see Table 2).

**TABLE 2:** Summary of evidence sources, therapeutic maturity, and key limitations of each intervention category.

Intervention category	Agent	Source of evidence	Therapeutic maturity	Key limitations and challenges
Mitochondrial antioxidants	SIRT1 activators	Ocular preclinical studies	Low (basic research)	Although SIRT1 is widely expressed in the cornea and trabecular meshwork, small-molecule activators generally exhibit poor ocular bioavailability; eye drops cannot effectively reach the retina; clinical application requires further validation
	Humanin	Ocular preclinical studies	Low (basic research)	Peptide molecules have a short half-life and rapid clearance *in vivo*, necessitating repeated administration; eye drops cannot penetrate the retina; intravitreal injection increases invasiveness and complication risk; clinical application requires further validation
	Sappanone A	Ocular preclinical studies	Low (basic research)	Poor water solubility of this natural small molecule limits penetration across the retinal barrier; effective dosage and therapeutic window remain undefined; not yet clinically applicable
Mitochondrial biogenesis enhancers	Resveratrol	Small-sample ophthalmic clinical studies	Moderate (clinical and basic research)	Clinical studies suggest therapeutic potential; however, large-scale trials are lacking, and results show significant heterogeneity; currently used only as an adjunctive nutritional exploration; expanded clinical research is needed
	Crocetin	Small-sample ophthalmic clinical studies	Moderate (clinical and basic research)	Small-sample trials in diabetic maculopathy suggest improved retinal function; however, the compound is lipophilic with limited ocular tissue enrichment; ocular pharmacokinetic and toxicity data are lacking; further pharmacological studies are required
	Carotenoids	Ophthalmic clinical studies	Moderate (clinical research)	Carotenoids (e.g., lutein, zeaxanthin) may delay disease progression but cannot reverse established photoreceptor damage
	NAD^+^ precursors	Ocular preclinical and clinical studies	Moderate (predominantly basic research, limited clinical studies)	Oral NAD^+^ precursors undergo rapid systemic metabolism; the effective concentration reaching the retina is limited; long-term clinical studies are lacking
	Human embryonic stem cells	Ocular preclinical and clinical studies	Moderate (predominantly basic research, limited clinical studies)	Complete clinical trial data are lacking; high cost
Mitochondrial dynamics regulators	Tacrolimus	Ocular preclinical and clinical studies	High (clinical research, alongside basic research)	Dosing concentrations and formulations vary across studies; large-scale long-term safety data are lacking
	Danluoning	Ocular preclinical studies	Low (basic research)	Complex multi-component formulation; active monomer not clearly defined; target mechanisms are difficult to elucidate
	Tanshinone IIa	Ocular preclinical studies	Low (basic research)	Highly lipophilic and poorly water-soluble, posing formulation challenges for eye drops; most animal studies use intraperitoneal or intravitreal injection, which differs substantially from clinical topical administration
	Apigenin	Ocular preclinical studies	Low (basic research)	Poor water solubility and low ocular bioavailability; eye drops cannot effectively reach the retina; most animal studies use preventive administration; human ocular safety and efficacy data are lacking
​	Vorinostat	Ocular preclinical studies	Low (basic research)	Significant systemic toxicity; unsuitable for long-term systemic use in ophthalmology; no clinical study data available
	Trichostatin A	Ocular preclinical studies	Low (basic research)	High toxicity and narrow therapeutic window; no mature ophthalmic formulation available; no clinical study data
Mitophagy inducers	Urolithin A	Ocular preclinical and clinical studies	Moderate (limited clinical studies, predominantly basic research)	Oral bioavailability is influenced by inter-individual gut microbiota variability; retinal tissue enrichment is limited; no mature ophthalmic formulation available
	Metformin	Ophthalmic clinical studies	Low (retrospective clinical studies)	Oral administration is unlikely to achieve therapeutic concentrations in the retina; intravitreal administration remains at the animal stage; ocular safety is unclear
	Notoginsenoside R1	Ocular preclinical studies	Low (basic research)	Limited water solubility and low bioavailability via topical administration; no clinical data available
	Fucoxanthin	Ocular preclinical studies	Low (basic research)	Lipophilic and chemically unstable, susceptible to oxidative degradation; no clinical data available
	PR-619	Ocular preclinical studies	Low (basic research)	Effective only via intravitreal injection; ineffective via eye drops; no mature ophthalmic formulation; no clinical data available
	Stigmasterol	Ocular preclinical studies	Low (basic research)	Poor water solubility; ocular delivery is the primary bottleneck; current studies are limited to a single model (diabetic retinal injury); no clinical data available

## 5 Conclusion

This review constructs an analytical framework based on cell type specificity to elucidate how mitochondrial metabolic characteristics transform into specific diseases under different pathological stresses. It systematically reviews the physiological roles of mitochondrial metabolism in key cell types of the eye and its central position in the pathogenesis of common blinding eye diseases. As a highly metabolic organ, the eye’s functionality relies on the coordinated work of various cell types, including RPECs, photoreceptor cells, scleral fibroblasts, ISMCs, TMCs, CECs, and RMECs. These cells are highly dependent on mitochondrial oxidative phosphorylation to support their specific physiological functions. Mitochondrial dysfunction, including ATP synthesis deficiency, excessive ROS production, cumulative mtDNA damage, imbalance in quality control networks, and disruption of dynamic homeostasis, serves as a common pathological basis driving the development of various ocular diseases, such as myopia, DR and glaucoma.

Cell type-specific mitochondrial metabolic dependency underpins the selective vulnerability of different eye diseases. RPECs, due to their high reliance on mitochondrial energy supply to sustain continuous phagocytic loads, have a critical role in the disintegration of the lysosome-mitochondrion axis during aging. Retinal microvascular endothelial cells are particularly sensitive to mitochondrial oxidative stress induced by high glucose, as evidenced by the metabolic memory phenomenon mediated by mtDNA, explaining the persistent progression of DR even after glycemic control. Scleral fibroblasts, lacking vascular supply, are highly sensitive to hypoxic microenvironments, leading to collagen synthesis insufficiency and accelerated degradation, resulting in axial elongation. TMCs, due to their continuous phagocytic burden and oxidative stress exposure, exhibit a high dependency on complex I function, with mtDNA damage directly inducing glaucoma-like phenotypes. RGCs are highly sensitive to mitochondrial dysfunction and quality control defects due to their axonal dependency on mitochondrial axoplasmic transport, with genetic factors such as OPTN further exacerbating their metabolic vulnerability. This cell type-specific metabolic vulnerability provides a unified theoretical framework for understanding the pathogenesis of different eye diseases and paves the way for precision interventions based on cellular metabolic characteristics.

Targeted mitochondrial therapeutic strategies are rapidly advancing from basic research to clinical applications, showing broad prospects for application. Mitochondrial antioxidants, mitochondrial biogenesis enhancers, mitochondrial dynamics regulators, and mitochondrial autophagy inducers improve mitochondrial function from various perspectives. Similarly, the response to mitochondrial interventions varies cell type-specifically in different eye diseases—RGCs are particularly sensitive to NAD^+^ precursor supplementation, RPECs respond more significantly to SIRT1 activators, TMCs have a good response to Drp1 inhibition, and retinal microvascular endothelial cells require restoring mitochondrial autophagy to block metabolic memory. Therefore, future research should focus on developing more cell type-specific mitochondrial-targeted delivery systems to achieve precise drug distribution at the cellular and organelle levels. By establishing patient metabolic subtyping through metabolic imaging and body fluid metabolomics, this will guide personalized treatment strategies. Additionally, rigorous multicenter randomized controlled trials should be conducted to validate the long-term safety and efficacy of mitochondrial-targeted drugs. The eye not only offers advantages for local drug administration and high-resolution live imaging but is also highly sensitive to mitochondrial damage, making it an ideal model organ for translating mitochondrial-targeted therapies from basic research to clinical applications. This could bring breakthroughs in intervention strategies for degenerative ocular diseases such as glaucoma, and DR, which are centered on mitochondrial dysfunction.

It is important to acknowledge that most of the mechanistic and therapeutic evidence discussed in this review is derived from animal models, which have inherent limitations. Species-specific differences in ocular anatomy, e.g., the presence or absence of a macula, retinal vascular architecture, and visual physiology may influence the translational relevance of experimental findings. For example, rodents lack a true macula, which limits their utility for modeling macular diseases such as AMD, and their retinal metabolism differs from that of primates in ways that may affect responses to mitochondrial interventions. Similarly, the lens and vitreous volumes, as well as the pharmacokinetics of intraocular drug delivery, vary substantially across species. These differences should be considered when extrapolating preclinical data to human ocular diseases, and wherever possible, findings should be validated in higher-order animal models or human tissue studies before clinical translation.

## References

[B1] Bhatti J. S. Bhatti G. K. Reddy P. H. (2017). Mitochondrial dysfunction and oxidative stress in metabolic disorders - a step towards mitochondria based therapeutic strategies. Biochimica biophysica acta Mol. basis Dis. 1863 (5), 1066–1077. 10.1016/j.bbadis.2016.11.010 27836629 PMC5423868

[B2] Brown E. E. DeWeerd A. J. Ildefonso C. J. Lewin A. S. Ash J. D. (2019). Mitochondrial oxidative stress in the retinal pigment epithelium (RPE) led to metabolic dysfunction in both the RPE and retinal photoreceptors. Redox Biology 24, 101201. 10.1016/j.redox.2019.101201 31039480 PMC6488819

[B3] Chaqour B. Rossman J. B. Meng M. Dine K. E. Ross A. G. Shindler K. S. (2025). SIRT1-based therapy targets a gene program involved in mitochondrial turnover in a model of retinal neurodegeneration. Sci. Reports 15 (1), 13585. 10.1038/s41598-025-97456-8 PMC12009334 40253451

[B4] Che S. Ma Y. Fu J. Zhou X. Cao J. (2026). Oxidative stress in diabetic retinopathy: metabolic triggers, molecular pathways and emerging antioxidant therapies. Int. Journal Biological Macromolecules 344 (Pt 2), 150272. 10.1016/j.ijbiomac.2026.150272 41539508

[B5] Chen M. Zhang Q. Wang S. Zheng F. (2023). Inhibition of diabetes-induced Drp1 deSUMOylation prevents retinal vascular lesions associated with diabetic retinopathy. Exp. Eye Research 226, 109334. 10.1016/j.exer.2022.109334 36435207

[B6] Chen C. Liu Q. Tang C. Rong Y. Zhao X. Li D. (2025). ZC3H11A mutations cause high myopia by triggering PI3K-AKT and NF-κB-mediated signaling pathway in humans and mice. eLife 12, RP91289. 10.7554/elife.91289.4 PMC12387752 40864167

[B7] Choudhury C. Gill M. K. McAleese C. E. Butcher N. J. Ngo S. T. Steyn F. J. (2024). The arylamine N-Acetyltransferases as therapeutic targets in metabolic diseases associated with mitochondrial dysfunction. Pharmacol. Reviews 76 (2), 300–320. 10.1124/pharmrev.123.000835 38351074

[B8] Chuang Y.-C. Chen S.-D. Hsu C.-Y. Chen S.-F. Chen N.-C. Jou S.-B. (2019). Resveratrol promotes mitochondrial biogenesis and protects against seizure-induced neuronal cell damage in the hippocampus following status epilepticus by activation of the PGC-1α signaling pathway. Int. Journal Molecular Sciences 20 (4), 998. 10.3390/ijms20040998 30823590 PMC6412811

[B9] Cimaglia G. Tribble J. R. Votruba M. Williams P. A. Morgan J. E. (2024). Oral nicotinamide provides robust, dose-dependent structural and metabolic neuroprotection of retinal ganglion cells in experimental glaucoma. Acta Neuropathol. Communications 12 (1), 137. 10.1186/s40478-024-01850-8 PMC11342512 39180087

[B10] Claassens N. J. Volpers M. Dos Santos VAPM van der Oost J. de Vos W. M. (2013). Potential of proton-pumping rhodopsins: engineering photosystems into microorganisms. Trends Biotechnology 31 (11), 633–642. 10.1016/j.tibtech.2013.08.006 24120288

[B11] Cueva Vargas J. L. Belforte N. Vidal-Paredes I. A. Dotigny F. Vande Velde C. Quintero H. (2026). Stress-induced mitochondrial fragmentation in endothelial cells disrupts blood-retinal barrier integrity causing neurodegeneration. Mol. Therapy The Journal Am. Soc. Gene Ther. 34 (1), 562–581. 10.1016/j.ymthe.2025.09.037 PMC12925807 40994007

[B12] Deliyanti D. Alrashdi S. F. Tan S. M. Meyer C. Ward K. W. de Haan J. B. (2018). Nrf2 activation is a potential therapeutic approach to attenuate diabetic retinopathy. Investigative Ophthalmology and Visual Science 59 (2), 815–825. 10.1167/iovs.17-22920 29411009

[B13] Gabella G. (2018). Mitochondria in smooth muscle cells of viscera. J. Smooth Muscle Research = Nihon Heikatsukin Gakkai Kikanshi 54 (0), 51–70. 10.1540/jsmr.54.51 30047517 PMC6060277

[B14] Gentle A. Liu Y. Martin J. E. Conti G. L. McBrien N. A. (2003). Collagen gene expression and the altered accumulation of scleral collagen during the development of high myopia. J. Biological Chemistry 278 (19), 16587–16594. 10.1074/jbc.M300970200 12606541

[B15] Gill K. P. Hung S. S. C. Sharov A. Lo C. Y. Needham K. Lidgerwood G. E. (2016). Enriched retinal ganglion cells derived from human embryonic stem cells. Sci. Reports 6, 30552. 10.1038/srep30552 27506453 PMC4978994

[B16] Gong W. Shen Z. Zhao L. Cheng T. Zhang B. Chen J. (2025). N-Acetyltransferase 2 inhibits myopia by maintaining mitochondrial metabolism in scleral fibroblasts. Res. Wash. DC 8, 0969. 10.34133/research.0969 PMC12569602 41169617

[B17] Harper A. R. Summers J. A. (2015). The dynamic sclera: extracellular matrix remodeling in normal ocular growth and myopia development. Exp. Eye Research 133, 100–111. 10.1016/j.exer.2014.07.015 25819458 PMC4379420

[B18] Hatou S. (2011). Hormonal regulation of Na+/K+-dependent ATPase activity and pump function in corneal endothelial cells. Cornea 30 (Suppl. 1), S60–S66. 10.1097/ICO.0b013e318227faab 21912233

[B19] He X. Li S. M. (2023). Gene-environment interaction in myopia. Ophthalmic Physiol. Opt. 43 (6), 1438–1448. 10.1111/opo.13206 37486033

[B20] He Y. Ge J. Tombran-Tink J. (2008). Mitochondrial defects and dysfunction in calcium regulation in glaucomatous trabecular meshwork cells. Investigative Ophthalmology and Visual Science 49 (11), 4912–4922. 10.1167/iovs.08-2192 18614807

[B21] He W. Tang P. Lv H. (2025). Targeting oxidative stress in diabetic retinopathy: mechanisms, pathology, and novel treatment approaches. Front. Immunology 16, 1571576. 10.3389/fimmu.2025.1571576 PMC12207546 40589740

[B22] Hostetler G. L. Ralston R. A. Schwartz S. J. (2017). Flavones: food sources, bioavailability, metabolism, and bioactivity. Adv. Nutrition (Bethesda, Md) 8 (3), 423–435. 10.3945/an.116.012948 28507008 PMC5421117

[B23] Hu X. Zhang J. Ma H. Lian W. Song W. Du C. (2024). The broad-spectrum deubiquitinating enzyme inhibitor PR-619 protects retinal ganglion cell and augments parkin-mediated mitophagy in experimental glaucoma. Sci. Reports 14 (1), 24654. 10.1038/s41598-024-75562-3 PMC11491476 39428410

[B24] Jeong Y. Davis C.-H. O. Muscarella A. M. Navarro H. H. Deshpande V. Moore L. G. (2025). Glaucoma-associated optineurin mutations increase transcellular degradation of mitochondria in a vertebrate optic nerve. eLife 14, e103844. 10.7554/elife.103844 PMC12599881 40795095

[B25] Jiang D. Chen F.-X. Zhou H. Lu Y.-Y. Tan H. Yu S.-J. (2020). Bioenergetic crosstalk between mesenchymal stem cells and various ocular cells through the intercellular trafficking of mitochondria. Theranostics 10 (16), 7260–7272. 10.7150/thno.46332 32641991 PMC7330858

[B26] Jiménez-Loygorri J. I. Villarejo-Zori B. Viedma-Poyatos Á. Zapata-Muñoz J. Benítez-Fernández R. Frutos-Lisón M. D. (2024). Mitophagy curtails cytosolic mtDNA-dependent activation of cGAS/STING inflammation during aging. Nat. Communications 15 (1), 830. 10.1038/s41467-024-45044-1 PMC10821893 38280852

[B27] Jiménez-Loygorri J. I. Benítez-Fernández R. Viedma-Poyatos Á. Zapata-Muñoz J. Villarejo-Zori B. Gómez-Sintes R. (2023). Mitophagy in the retina: viewing mitochondrial homeostasis through a new lens. Prog. Retinal Eye Research 96, 101205. 10.1016/j.preteyeres.2023.101205 37454969

[B28] Jiménez-Loygorri J. I. Viedma-Poyatos Á. Gómez-Sintes R. Boya P. (2024). Urolithin A promotes p62-dependent lysophagy to prevent acute retinal neurodegeneration. Mol. Neurodegeneration 19 (1), 49. 10.1186/s13024-024-00739-3 PMC11186080 38890703

[B29] Kanda H. Kaneda T. Kato A. Yogo T. Harada Y. Hara Y. (2016). Aerobic metabolism on muscle contraction in Porcine Iris sphincter. J. Veterinary Medical Science 78 (11), 1673–1676. 10.1292/jvms.16-0175 27506577 PMC5138420

[B30] Kang E. Y.-C. Liu P.-K. Wen Y.-T. Quinn P. M. J. Levi S. R. Wang N.-K. (2021). Role of oxidative stress in ocular diseases associated with retinal ganglion cells degeneration. Antioxidants Basel, Switz. 10 (12), 1948. 10.3390/antiox10121948 PMC8750806 34943051

[B31] Kanow M. A. Giarmarco M. M. Jankowski C. S. Tsantilas K. Engel A. L. Du J. (2017). Biochemical adaptations of the retina and retinal pigment epithelium support a metabolic ecosystem in the vertebrate eye. Elife 6, e28899. 10.7554/eLife.28899 28901286 PMC5617631

[B32] Kassab A. Piwowar A. (2012). Cell oxidant stress delivery and cell dysfunction onset in type 2 diabetes. Biochimie 94 (9), 1837–1848. 10.1016/j.biochi.2012.01.020 22333037

[B33] Kim D. Roy S. (2020). Effects of diabetes on mitochondrial morphology and its implications in diabetic retinopathy. Investigative Ophthalmology and Visual Science 61 (10), 10. 10.1167/iovs.61.10.10 PMC7441301 32756920

[B34] Kowluru R. A. (2013). Mitochondria damage in the pathogenesis of diabetic retinopathy and in the metabolic memory associated with its continued progression. Curr. Med. Chem. 20 (26), 3226–3233. 10.2174/09298673113209990029 23745551 PMC10280299

[B35] Kowluru R. A. (2023). Cross talks between oxidative stress, inflammation and epigenetics in diabetic retinopathy. Cells 12 (2), 300. 10.3390/cells12020300 PMC9857338 36672234

[B36] Kowluru R. A. Alka K. (2023). Mitochondrial quality control and metabolic memory phenomenon associated with continued progression of diabetic retinopathy. Int. Journal Molecular Sciences 24 (9), 8076. 10.3390/ijms24098076 PMC10179288 37175784

[B37] Kowluru R. A. Kowluru A. Mishra M. Kumar B. (2015). Oxidative stress and epigenetic modifications in the pathogenesis of diabetic retinopathy. Prog. Retin Eye Res. 48, 40–61. 10.1016/j.preteyeres.2015.05.001 25975734 PMC6697077

[B38] Kowluru R. A. Mohammad G. Kumar J. (2024). Impaired removal of the damaged mitochondria in the metabolic memory phenomenon associated with continued progression of diabetic retinopathy. Mol. Neurobiol. 61 (1), 188–199. 10.1007/s12035-023-03534-1 PMC10791911 37596436

[B39] Li Y. Tian L. Li S. Chen X. Lei F. Bao J. (2024). Disrupted mitochondrial transcription factor A expression promotes mitochondrial dysfunction and enhances ocular surface inflammation by activating the absent in melanoma 2 inflammasome. Free Radical Biology and Medicine 222, 106–121. 10.1016/j.freeradbiomed.2024.05.032 38797339

[B40] Lin X. Lei Y. Pan M. Hu C. Xie B. Wu W. (2024). Augmentation of scleral glycolysis promotes myopia through histone lactylation. Cell Metab. 36 (3), 511. 10.1016/j.cmet.2023.12.023 38232735

[B41] Lindsay K. J. Du J. Sloat S. R. Contreras L. Linton J. D. Turner S. J. (2014). Pyruvate kinase and aspartate-glutamate carrier distributions reveal key metabolic links between neurons and glia in retina. Proc. Natl. Acad. Sci. U. S. A. 111 (43), 15579–15584. 10.1073/pnas.1412441111 25313047 PMC4217417

[B42] Liu Y. Bu Q. Hu D. Chen C. Zhu J. Zhou Q. (2024). NAD(+) supplementation improves mitochondrial functions and normalizes glaucomatous trabecular meshwork features. Exp. Cell Research 440 (1), 114137. 10.1016/j.yexcr.2024.114137 38897410

[B43] Lu S. Y. He Z. Z. Xu J. X. Yang C. Chen L. J. Gong B. (2019). Association of polymorphisms at the SIX1-SIX6 locus with primary open-angle glaucoma. Investigative Ophthalmology and Visual Science 60 (8), 2914–2924. 10.1167/iovs.18-26489 31284308

[B44] Lugassy Y. Berent E. Tarony L. Jeries S. Ziv T. Savion N. (2025). HDAC inhibition protects RPE cells from oxidative stress via enhanced mitochondrial fusion, cytoskeletal repair, and Nrf-2 activation. Free Radical Biology and Medicine 240, 59–70. 10.1016/j.freeradbiomed.2025.08.007 40816649

[B45] Lv K. Ying H. Hu G. Hu J. Jian Q. Zhang F. (2022). Integrated multi-omics reveals the activated retinal microglia with intracellular metabolic reprogramming contributes to inflammation in STZ-Induced early diabetic retinopathy. Front. Immunol. 13, 942768. 10.3389/fimmu.2022.942768 PMC9479211 36119084

[B46] Ma H. Hu X. Zhang J. Lian W. Wang D. Zhang L. (2025). Fucoxanthin protects retinal ganglion cells and regulates Parkin-mediated mitophagy in experimental glaucoma. BMJ Open Ophthalmology 10 (1), e002126. 10.1136/bmjophth-2024-002126 PMC12374660 40841125

[B47] McWilliams T. G. Prescott A. R. Villarejo-Zori B. Ball G. Boya P. Ganley I. G. (2019). A comparative map of macroautophagy and mitophagy in the vertebrate eye. Autophagy 15 (7), 1296–1308. 10.1080/15548627.2019.1580509 30786807 PMC6613837

[B48] Mohammad S. I. Vasudevan A. Nuseir M. Baig M. R. Yakhshieva Z. Turakulov R. (2026). The redox code of diabetic retinopathy: decoding metabolic memory, predicting fate, and targeting heterogeneity. Exp. Eye Research 272, 111200. 10.1016/j.exer.2026.111200 42601009

[B49] Nashine S. Kenney M. C. (2024). Effects of humanin G (HNG) on angiogenesis and neurodegeneration markers in Age-related macular degeneration (AMD). Mitochondrion 74, 101818. 10.1016/j.mito.2023.11.001 38029849

[B50] Nentwich M. M. Ulbig M. W. (2015). Diabetic retinopathy - ocular complications of diabetes mellitus. World Journal Diabetes 6 (3), 489–499. 10.4239/wjd.v6.i3.489 25897358 PMC4398904

[B51] Nian S. Lo A. C. Y. Mi Y. Ren K. Yang D. (2021). Neurovascular unit in diabetic retinopathy: pathophysiological roles and potential therapeutical targets. Eye Vision Lond. Engl. 8 (1), 15. 10.1186/s40662-021-00239-1 PMC8088070 33931128

[B52] Otsu W. Yako T. Sugisawa E. Nakamura S. Tsusaki H. Umigai N. (2022). Crocetin protects against mitochondrial damage induced by UV-A irradiation in corneal epithelial cell line HCE-T cells. J. Pharmacological Sciences 150 (4), 279–288. 10.1016/j.jphs.2022.10.005 36344051

[B53] Pan L. Wu J. Wang N. (2024). Association of gene polymorphisms with normal tension glaucoma: a systematic review and meta-analysis. Genes 15 (4), 491. 10.3390/genes15040491 PMC11050218 38674425

[B54] Qian S. Qian Y. Huo D. Wang S. Qian Q. (2019). Tanshinone IIa protects retinal endothelial cells against mitochondrial fission induced by methylglyoxal through glyoxalase 1. Eur. Journal Pharmacology 857, 172419. 10.1016/j.ejphar.2019.172419 31136758

[B55] Ren Y. Yang X. Luo Z. Wu J. Lin H. (2022a). HIF-1α aggravates pathologic myopia through the miR-150-5p/LAMA4/p38 MAPK signaling axis. Mol. Cellular Biochemistry 477 (4), 1065–1074. 10.1007/s11010-021-04305-z 35034256

[B56] Ren J. Zhang S. Pan Y. Jin M. Li J. Luo Y. (2022b). Diabetic retinopathy: involved cells, biomarkers, and treatments. Front. Pharmacology 13, 953691. 10.3389/fphar.2022.953691 PMC9396039 36016568

[B57] Ridley R. B. Amontree A. C. Lewin A. S. Ildefonso C. J. (2025). Mitochondrial DNA damage in the retinal pigmented epithelium (RPE) and its role in RPE pathobiology. Adv. Experimental Medicine Biology 1468, 375–379. 10.1007/978-3-031-76550-6_62 39930225

[B58] Ryu Y. Seo J. H. Kim H. S. Nam Y. J. Bo Noh K. Oh S.-H. (2024). COL8A2 activation enhances function of corneal endothelial cells through HIPPO signaling/mitochondria pathway. Matrix Biology Journal Int. Soc. Matrix Biol. 134, 119–131. 10.1016/j.matbio.2024.10.001 39395654

[B59] Saccà S. C. Pulliero A. Izzotti A. (2015). The dysfunction of the trabecular meshwork during glaucoma course. J. Cellular Physiology 230 (3), 510–525. 10.1002/jcp.24826 25216121

[B60] Saccà S. C. Gandolfi S. Bagnis A. Manni G. Damonte G. Traverso C. E. (2016). From DNA damage to functional changes of the trabecular meshwork in aging and glaucoma. Ageing Research Reviews 29, 26–41. 10.1016/j.arr.2016.05.012 27242026

[B61] Shaito A. Al-Mansoob M. Ahmad S. M. S. Haider M. Z. Eid A. H. Posadino A. M. (2023). Resveratrol-mediated regulation of mitochondria biogenesis-associated pathways in neurodegenerative diseases: molecular insights and potential therapeutic applications. Curr. Neuropharmacology 21 (5), 1184–1201. 10.2174/1570159X20666221012122855 PMC10286596 36237161

[B62] Shan M. Fan T.-J. (2016). Cytotoxicity of carteolol to human corneal epithelial cells by inducing apoptosis *via* triggering the Bcl-2 family protein-mediated mitochondrial pro-apoptotic pathway. Toxicol. Vitro An International Journal Published Association BIBRA 35, 36–42. 10.1016/j.tiv.2016.05.008 27216471

[B63] She M. Li T. Shi W. Li B. Zhou X. (2022). AREG is involved in scleral remodeling in form-deprivation myopia via the ERK1/2-MMP-2 pathway. FASEB Journal Official Publication Fed. Am. Soc. Exp. Biol. 36 (5), e22289. 10.1096/fj.202200132R 35436023

[B64] Shen H. Ding C. Yuan S. Pan T. Li D. Li H. (2020). Vitamin C- and valproic acid-induced fetal RPE stem-like cells recover retinal degeneration via regulating SOX2. Mol. Therapy The Journal Am. Soc. Gene Ther. 28 (7), 1645–1657. 10.1016/j.ymthe.2020.04.008 32353323 PMC7335738

[B65] Shi S. Ou X. Liu C. Wen H. Ke J. (2025). Research progress of HIF-1a on immunotherapy outcomes in immune vascular microenvironment. Front. Immunology 16, 1549276. 10.3389/fimmu.2025.1549276 PMC11839635 39981236

[B66] Song Y. Wang M. Zhao S. Tian Y. Zhang C. (2022). Matrine promotes mitochondrial biosynthesis and reduces oxidative stress in experimental optic neuritis. Front. Pharmacology 13, 936632. 10.3389/fphar.2022.936632 PMC9552203 36238552

[B67] Sun Y. Li Z.-Z. Yang J. Sha Y.-R. Hou X.-Y. Fu H. (2024). Molecular mechanism of hypoxia and alpha-ketoglutaric acid on collagen expression in scleral fibroblasts. Int. Journal Ophthalmology 17 (10), 1780–1790. 10.18240/ijo.2024.10.03 PMC11422372 39430015

[B68] Sun H. Li B. Gu Y. Li F. Di G. Chen P. (2025). Imbalanced mitochondrial homeostasis in ocular diseases: unique pathogenesis and targeted therapy. Exp. Eye Research 260, 110632. 10.1016/j.exer.2025.110632 40930272

[B69] Sun Y. Sun Y. Wang M. Si Z. Zhang Z. Zheng Y. (2026). Chronic stress-induced steroids mediate mitochondrial fission and fibrosis in the trabecular meshwork via the MIEF1-MAOA complex. Free Radical Biology and Medicine 246, 316–333. 10.1016/j.freeradbiomed.2026.01.037 41579974

[B70] Tham Y. C. Li X. Wong T. Y. Quigley H. A. Aung T. Cheng C. Y. (2014). Global prevalence of glaucoma and projections of glaucoma burden through 2040: a systematic review and meta-analysis. Ophthalmology 121 (11), 2081–2090. 10.1016/j.ophtha.2014.05.013 24974815

[B71] Tong Y. Zhang Z. Wang S. (2022). Role of mitochondria in retinal pigment epithelial aging and degeneration. Front. Aging 3, 926627. 10.3389/fragi.2022.926627 PMC9337215 35912040

[B72] Toppila M. Ranta-Aho S. Kaarniranta K. Hytti M. Kauppinen A. (2024). Metformin alleviates inflammation and induces mitophagy in human retinal pigment epithelium cells suffering from mitochondrial damage. Cells 13 (17), 1433. 10.3390/cells13171433 PMC11394619 39273005

[B73] Van Bergen N. J. Crowston J. G. Craig J. E. Burdon K. P. Kearns L. S. Sharma S. (2015). Measurement of systemic mitochondrial function in advanced primary open-angle glaucoma and leber hereditary optic neuropathy. PloS One 10 (10), e0140919. 10.1371/journal.pone.0140919 26496696 PMC4619697

[B74] Venkatesan A. Bernstein A. M. (2025). Protein misfolding and mitochondrial dysfunction in glaucoma. Front. Cell Developmental Biology 13, 1595121. 10.3389/fcell.2025.1595121 PMC12083186 40385286

[B75] Visalli F. Cappellani F. Gagliano G. Spinello A. Avitabile A. Cannizzaro L. (2026). Mitochondrial resilience in glaucoma: targeting NAD(+) metabolism and oxidative stress in retinal ganglion cell degeneration with nicotinamide riboside and berberine: preliminary clinical evidence. Dis. Basel, Switz. 14 (2), 56. 10.3390/diseases14020056 PMC12939981 41745094

[B76] Vives-Bauza C. Anand M. Shiraz A. K. Magrane J. Gao J. Vollmer-Snarr H. R. (2008). The age lipid A2E and mitochondrial dysfunction synergistically impair phagocytosis by retinal pigment epithelial cells. J. Biological Chemistry 283 (36), 24770–24780. 10.1074/jbc.M800706200 18621729 PMC2529005

[B77] Wahyuningsih E. Wigid D. Dewi A. Moehariadi H. Sujuti H. Anandita N. (2022). The effect of citicoline on the expression of matrix Metalloproteinase-2 (MMP-2), transforming growth Factor-β1 (TGF-β1), and Ki-67, and on the thickness of scleral tissue of rat myopia model. Biomedicines 10 (10), 2600. 10.3390/biomedicines10102600 PMC9599282 36289864

[B78] Wang Y. Liu S. Huang Q. Cheng Y. (2025a). Mfn2 regulates scleral remodeling in myopia by maintaining endoplasmic reticulum homeostasis in scleral fibroblasts. Investigative Ophthalmology and Visual Science 66 (14), 40. 10.1167/iovs.66.14.40 PMC12633772 41247129

[B79] Wang Q. Zhang P. Ji F. Li Y. Lu P. (2025b). Stigmasterol improves diabetic retinopathy by inhibiting VAMP7-mediated autophagy/mitophagy. Biochem. Biophysical Research Communications 785, 152653. 10.1016/j.bbrc.2025.152653 40997581

[B80] Wang X. Gui S. Liu X. Huang Z. Liu H. Gao J. (2026). Sappanone A alleviates high glucose-induced retinal oxidative injury and mitochondrial dysfunction via SIRT7/NRF2 pathway. J. Ethnopharmacology 356, 120774. 10.1016/j.jep.2025.120774 41135616

[B81] Wareham L. K. Liddelow S. A. Temple S. Benowitz L. I. Di Polo A. Wellington C. (2022). Solving neurodegeneration: common mechanisms and strategies for new treatments. Mol. Neurodegeneration 17 (1), 23. 10.1186/s13024-022-00524-0 PMC8935795 35313950

[B82] Wen X. Yang N. Gu C. Zhang Y. Hao Y. (2025a). Zeaxanthin improves myopia by regulating the HIF-1α-glycolysis signaling pathway. Exp. Eye Research 259, 110557. 10.1016/j.exer.2025.110557 40738388

[B83] Wen B. Li H. Tao H. Ren H. Ma B. Shi M. (2025b). Regulatory effects of the Wnt7b/β-Catenin/MMP-2 signaling pathway on scleral stiffness in Guinea pigs with form-deprivation myopia. Investigative Ophthalmology and Visual Science 66 (5), 19. 10.1167/iovs.66.5.19 PMC12068525 40338179

[B84] Whitehead M. Wickremasinghe S. Osborne A. Van Wijngaarden P. Martin K. R. (2018). Diabetic retinopathy: a complex pathophysiology requiring novel therapeutic strategies. Expert Opin. Biol. Ther. 18 (12), 1257–1270. 10.1080/14712598.2018.1545836 30408422 PMC6299358

[B85] Wu H. Chen W. Zhao F. Zhou Q. Reinach P. S. Deng L. (2018). Scleral hypoxia is a target for myopia control. Proc. Natl. Acad. Sci. U. S. A. 115 (30), E7091–E7100. 10.1073/pnas.1721443115 29987045 PMC6064999

[B86] Wu W. Su Y. Hu C. Tao H. Jiang Y. Zhu G. (2022). Hypoxia-induced scleral HIF-2α upregulation contributes to rises in MMP-2 expression and myopia development in mice. Investigative Ophthalmology and Visual Science 63 (8), 2. 10.1167/iovs.63.8.2 PMC9279925 35802383

[B87] Wu J. Zhang D. Liu H. Li J. Li T. Wu J. (2024). Neuroprotective effects of apigenin on retinal ganglion cells in ischemia/reperfusion: modulating mitochondrial dynamics in *in* vivo and in vitro models. J. Translational Medicine 22 (1), 447. 10.1186/s12967-024-05260-1 PMC11089678 38741132

[B88] Yang R.-Z. Wang Y. Wu Z. Luo Y. Wang D.-D. Zou Y. (2025). Ambient light alleviates retinal neurodegeneration in mice by powering mitochondria via the engineered optoenergetic rhodopsin. Signal Transduction Targeted Therapy 10 (1), 355. 10.1038/s41392-025-02450-1 PMC12572652 41162360

[B89] Yang L. Chen S. Ge J. Chen Z. Chen K. Li J. (2026). Mitochondria in corneal physiology and pathology: a mechanistic perspective. Prog. Retinal Eye Research 110, 101432. 10.1016/j.preteyeres.2025.101432 41448387

[B90] Yu Q. Zhou J.-B. (2022). Scleral remodeling in myopia development. Int. Journal Ophthalmology 15 (3), 510–514. 10.18240/ijo.2022.03.21 PMC8907044 35310066

[B91] Yuan Z. Tian Y. Zhang C. Wang M. Xie J. Wang C. (2023). Integration of systematic review, lipidomics with experiment verification reveals abnormal sphingolipids facilitate diabetic retinopathy by inducing oxidative stress on RMECs. Biochimica biophysica acta Mol. Cell Biol. lipids 1868 (11), 159382. 10.1016/j.bbalip.2023.159382 37659619

[B92] Zhang X. Feng Y. Wang Y. Wang J. Xiang D. Niu W. (2018). Resveratrol ameliorates disorders of mitochondrial biogenesis and dynamics in a rat chronic ocular hypertension model. Life Sciences 207, 234–245. 10.1016/j.lfs.2018.06.010 29894715

[B93] Zhang Q. Chen J. Li H. Yang J. Huang J. Liu B. (2025). Dehydroepiandrosterone prevents collagen loss by regulating HIF-1α expression and mitophagy in hypoxic human scleral fibroblasts. Am. Journal Pathology 195 (12), 2451–2462. 10.1016/j.ajpath.2025.07.018 40946797

[B94] Zhang N. Han G. Hao R. (2026). The role of circadian rhythms in the pathogenesis of myopia. Front. Physiol. 17, 1797489. 10.3389/fphys.2026.1797489 PMC13078985 41994054

[B95] Zhao F. Zhang D. Zhou Q. Zhao F. He M. Yang Z. (2020). Scleral HIF-1α is a prominent regulatory candidate for genetic and environmental interactions in human myopia pathogenesis. EBioMedicine 57, 102878. 10.1016/j.ebiom.2020.102878 32652319 PMC7348000

[B96] Zhou P. Xie W. Meng X. Zhai Y. Dong X. Zhang X. (2019). Notoginsenoside R1 ameliorates diabetic retinopathy through PINK1-Dependent activation of mitophagy. Cells 8 (3), 213. 10.3390/cells8030213 30832367 PMC6468581

